# Long Term and Large-Scale Continuous Studies on Zinc(II) Sorption and Desorption on Hybrid Pectin-Guar Gum Biosorbent

**DOI:** 10.3390/polym11010096

**Published:** 2019-01-08

**Authors:** Agata Jakóbik-Kolon, Joanna Bok-Badura, Andrzej Milewski, Krzysztof Karoń

**Affiliations:** Faculty of Chemistry, Silesian University of Technology, Krzywoustego 6, 44-100 Gliwice, Poland; joanna.bok-badura@polsl.pl (J.B.-B.); andrzej.milewski@polsl.pl (A.M.); krzysztof.karon@polsl.pl (K.K.)

**Keywords:** pectin-based biosorbent, column studies, large-scale, zinc removal

## Abstract

Pectin-guar gum biosorbent was tested for zinc(II) ions removal in column process. Sorption–desorption experiments were performed in laboratory and at larger scale. The breakthrough and elution curves were obtained for various conditions. The Bed Depth Service Time model was tested for utility in data estimation. Possibility of sorbent reuse and its lifetime was examined in 20 repeated sorption–desorption cycles. Finally, tests were repeated for real wastewater from galvanizing plant, giving satisfactory results. The effectiveness of Zn(II) sorption happened to be dependent on process parameters; tests have proved that it increased with increasing bed height and with decreasing flow rate or grain size. For an initial zinc concentration of 30 mg/L, even 2096 mL of zinc solution could be purified in small scale experiment (2 g of fine grain sorbent and flow rate 60 mL/h) or 5900 L in large-scale (16 kg of large grain sorbent and flow rate 45 L/h). This allowed for 40-fold or 49-fold zinc increases in concentration in one sorption–desorption cycle. The most successful results are meant that at least 20 sorption–desorption cycles could be performed on one portion of biosorbent without loss of its effectiveness, large-scale tests for real wastewater from galvanizing plant gave satisfactory results, and that the form and mechanical stability of our sorbent is suitable for column usage with flow rates applicable in industry.

## 1. Introduction

Zinc and its compounds are usually considered as important essential elements for human and other living organisms. Zinc does not arouse fear like other heavy metals e.g., mercury or cadmium, thus may be downplayed. However, its excess is harmful to e.g., the nervous and respiratory systems and the gastrointestinal tract [1] and may cause disorders in the environment e.g., in soil [2]. Therefore it is a real threat, especially given that worldwide production of zinc is enormous (13 million tons per year). Wastewater containing zinc is mainly from industry (e.g., construction, automotive, hardware, electrical, medical, cosmetic, and paint industries) or agriculture origin, but mine water from zinc ore mining may be also the source of this element [3]. The zinc content in wastewater is usually rather low (below 100 mg/L [3,4,5,6,7]), but it still exceeds the limits permitted by law for wastewater introduced into the environment (1.5–2.61 mg/L [8,9,10]). One of the most suitable method for purification of such diluted wastewater from heavy metal ions is sorption. Among others, biosorbents are widely investigated as cheap and biodegradable materials (e.g., algae, citrus peels, rice husk, sawdust, coffee and tea grounds, yeasts, nut shells, bacteria, etc. [11,12,13,14,15,16,17,18,19,20,21]). Most research concerns the batch (periodical) process; column studies, though more advantageous, are rather rare, probably due to inapplicable physical form of used biosorbents. Fine and irregular particles may clog the column and relatively high pressure has to be applied to flow the solution through the bed [22]. Occasionally pilot-scale studies are presented that indicate that the problem grows with the scale-up of the process [23]. However, column processes are more desirable in the industry as they are less space-consuming, less time-consuming, and easier to automation than periodical ones. Other important factors are possibility of sorbent reuse [21] and its lifetime—they help define the economical aspect of biosorbent usage. The examination considering the desorption of the pollutant as well as biosorbent reuse and lifetime are often overlooked [22].

In our previous studies we have presented batch studies on the pectin-based biosorbents of zinc sorption capacities similar or rather higher to other biosorbents presented in the literature [24]. They were of the round beads form, which seemed to be suitable for column application. Additionally, the possibility of stripping the zinc(II) ions from studied materials by diluted acid solutions was confirmed.

Therefore, the aim of these investigations as the next stage of studies on pectin-based biosorbents was to test this material for zinc ions removal in column processes, firstly in small scale and then in semi-technical (3200 times greater) scale. For this purpose, a pectin-based biosorbent with guar gum additive was selected. This additive did not change the sorbent properties significantly but since is cheaper than pectin, significantly reduce the price of the final biosorbent.

## 2. Materials and Methods 

### 2.1. Materials

Amide pectin (type NECJ A20, amidation degree 19.9% and esterification degree 30.2%) was purchased from C&G Sp. z o.o. (Jasło, Poland). Guar gum was supplied by Agnex (Białystok, Poland). The following reagents were also used: concentrated nitric, sulfuric and hydrochloric acids (all “Suprapur” from Merck, Darmstadt, Germany), sodium hydroxide, calcium chloride (Avantor, Gliwice, Poland), zinc nitrate (Merck, Darmstadt, Germany), and standard solution of zinc (1000 mg/L, Merck, Darmstadt, Germany). Deionized water for small (laboratory) scale experiments and analytical purpose was prepared using Millipore Elix 10 system (Milipore SAS, Molsheim, France). For large-scale experiments, pure water of conductivity about 4 μS was purchased from Delta Karol Chmiel (Żory, Poland). 

The fine grain of pectin-guar gum biosorbent, which was previously obtained and characterized [24,25] was used. The larger grain of hybrid pectin-based sorbent containing guar gum (the same ratio P:G = 1:0.5) for large-scale studies was obtained in the same way, but silicon tubes of larger dimension (2 mm instead of 1 mm) for polysaccharide solution instilling were used. As the result amber colored beads of xerogel of diameter ca. 2 mm (1.6–2.6 mm, dry form, moisture content ca. 10%) were obtained.

The wastewater from galvanizing plant was kindly supplied by Nycz Intertrade Sp. z o.o. (Niepołomice, Poland). To avoid transportation of large amounts of wastewater, we have used the original concentrated weakly acidic bath for zinc electroplating which was then diluted with deionized water to final Zn concentration of 30 mg/L (as in the model solution) in order to simulate rinse water from the galvanizing plant. The composition of the bath was as follows; zinc chloride (~40 g/L), potassium chloride (~100 g/L), boric acid (~5 g/L), and a few additional components e.g., brightener, surfactants, etc. The exact composition of received wastewater is covered by trade secret. The pH of obtained final rinse water was 6.2. 

### 2.2. Analytical Methods and Apparatus

Concentration of zinc was determined by means of ICP-AES method (ICP atomic emission spectrometer Varian 710-ES, Varian, Palo Alto, CA, USA). Calibration curve method (Zn concentration range: 0.1–10 mg/L) was used. Biosorbents were analyzed utilizing scanning electron microscopy (Phenom Pro Desktop SEM, Eindhoven, The Netherlands) with an EDS detector preceded by lyophilization of swollen beads (Christ Alpha 1-2 LDplus, Martin Christ Gefriertrocknungsanlagen GmbH, Osterode am Harz, Germany). The thermostated shaker (Incu-Shaker, Benchmark, Sayreville, NJ, USA) was used for polysaccharide solutions preparation. Peristaltic pumps (Ismatec Reglo Digital, Cole-Parmer GmbH, Wertheim, Germany) were used for pumping the solution through the column in small scale studies, as well as for polysaccharide solution instilling into cold CaCl_2_ during sorbent preparation. The authors designed a large-scale column (internal diameter 240 mm, height 130 cm, volume ~60 L, PVC, transparent) equipped with peristaltic pumps, proper valves, and connectors (enabling drop-fed and reverse-fed column work); the control system as well as automatic sampling station was supplied by PPU Prokal Sp. z o.o. (Gliwice, Poland).

### 2.3. General Procedure for Laboratory Scale Column Sorption Studies

The glass column (internal diameter 15 mm) was loaded gradually with wet biosorbent and water to prevent trapping of air bubbles between the beads. The column was corked with rubber stopper equipped with pipe connected with feed solution. The feed solution (zinc(II) ions solution of initial concentration ~30 mg/L and pH = 6) was passed through the column (drop-fed system) at constant flow rate using peristaltic pump. The effluent from the column was collected into fractions of 20–100 mL and analyzed using ICP-AES method for zinc content.

The breakthrough curves were obtained by plotting ion concentration (c_Zn_ (mg/L)) or its change (fraction of initial concentration (c/c_0_)) against the volume of collected effluent (V (mL)).

The conditions for particular experiments were as follows

- for whole breakthrough curve: mass of sorbent (dry form): 1 g; flow rate: 30 mL/h;
- studies on effect of flow rate: mass of sorbent (dry form): 2 g; flow rate: 60 mL/h or 120 mL/h;
- studies on effect of bed height: mass of sorbent (dry form): 1 g (2.9 cm), 2 g (5.7 cm), or 4 g (11.2 cm); flow rate: 60 mL/h;
- studies on effect of grain size: mass of sorbent (dry form): small grain 4 g (11.2 cm) and large grain 5 g (11.2) cm; flow rate: 60 mL/h.

### 2.4. General Procedure for Laboratory Scale Column Desorption Studies

The biosorbent was loaded with Zn(II) ions according to the sorption general procedure (Section 2.3). Next, the acid solution (0.1 M HNO_3_) was passed through the column at constant flow rate using a peristaltic pump. The effluent from the column was collected into fractions of 10 mL and analyzed using ICP-AES method for zinc content. The dependence of concentration of zinc(II) ions in collected fractions (c_Zn_ (mg/L)) on volume of the collected effluent (V (mL)) was performed as elution curve. 

The conditions for particular experiments were as follows

- studies on effect of flow rate: mass of sorbent (dry form): 1 g; flow rate: 6 mL/h or 12 mL/h;
- studies on effect of bed height: mass of sorbent (dry form): 1 g or 2 g; flow rate: 12 mL/h.

Additionally of 20 cycles of sorption–desorption were performed using 5 g of large grain biosorbent (bed height 11.2 cm) and flow rate 60 mL/h. 

### 2.5. General Procedure for Large-Scale Column Sorption and Desorption Studies 

The transparent PVC column (internal diameter 240 mm, height 130 cm) was loaded with 16 kg (mass of dry form, ~55 L of wet form, bed height 120 cm) of swollen biosorbent and water to prevent trapping of air bubbles between the beads. The column was closed with cover, fixed with screws, and the feed solution (zinc(II) ions solution of initial concentration ~30 mg/L and pH = 6) was passed through the column using a peristaltic pump at constant flow rate. The construction of the column and its instrumentation enabled drop and reverse-fed column work. The zinc solution was prepared in IBC containers (volume of 1000 L) and the containers were connected with sorption station with transparent reinforced PVC tubes of diameter 30 mm (4 valves were available to connect 4 IBC containers simultaneously). The samples of effluent were taken every X minutes during the process using an automatic sampling station, where X may be individually set (in this studies ranged from 20–80 min depending on flow rate or necessity). The working apparatus is shown in Video S1. Samples were analyzed using ICP-AES method. Next the breakthrough curves were obtained (Section 2.3). 

The conditions for particular experiments were as follows

- studies on effect of flow rate: flow rate: 45 L/h or 200 L/h
- studies on zinc(II) ions removal from galvanizing plant wastewater: Zn initial concentration ~30 mg/L, pH ~6, flow rate: 200 L/h.

The biosorbent loaded with Zn(II) ions in the sorption process was then used in desorption studies. For this purpose the acid solution (0.1 M HNO_3_ or 0.05 M H_2_SO_4_) was passed through the column at constant flow rate of 30 L/h. The sampling of effluent and its analysis were the same as in the case of sorption studies. Next the elution curves were obtained (Section 2.4).

All column experiments were duplicated (small scale studies—two parallel columns— large-scale—two consecutive experiments on the same column) and all points for particular results were placed in the figures instead of average values.

The BDST (Bed Depth Service Time) model for some results prediction was used [26]:

t = (N_0_/(c_0_ × F)) × Z − (1/(K_a_c_0_)) × ln(c_0_/c_t_ − 1) (1)

where:

t—time (h) of obtaining in the effluent concentration c_t_ (mg/L)
N_0_—adsorption capacity (mg/L)
F—linear flow rate (cm/h)
K_a_—rate constant in BDST model (L/(mg × h))
c_0_—initial concentration of zinc(II) ions (mg/L)
Z—bed height (cm).

## 3. Results and Discussion

### 3.1. Biosorbent Preparation

The biosorbent for the large-scale study was needed in a huge amount in comparison to laboratory scale; therefore, some modifications of its preparation process had to be made. The instilling of the polysaccharides solution into cold CaCl_2_ solution using peristaltic pump equipped with tubes of 1 mm diameter was very slow. Additionally, after 2–3 h of instilling, the tubes were dilated thus further significant deceleration of instilling rate was observed. This was avoided when tubes of 2 mm diameter were used and instilling rate was also definitely higher. This enabled the preparation of 17 kg of pectin-based biosorbent in laboratory conditions in a reasonable time. Obtained biosorbent grains of app. 2 mm diameter (dry form) were used firstly in small and then in large column studies.

### 3.2. Column Studies—Laboratory Scale

**Figure 1:**
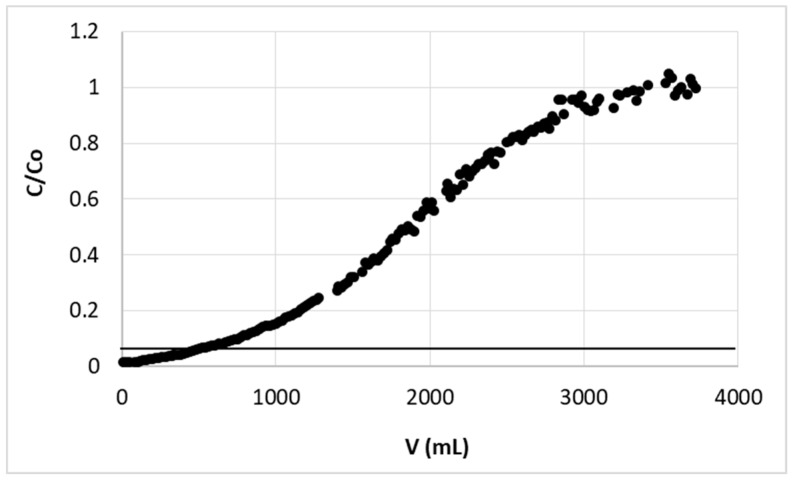
Zinc(II) ions breakthrough curve on hybrid pectin-based biosorbent. Mass of sorbent (dry form, fine grain): 1 g; flow rate: 30 mL/h; initial concentration of Zn: 30 mg/L; pH = 6.

The fine grain pectin-based biosorbent, used previously in batch studies [24], was tested in column experiments for zinc ions removal. The column processes in comparison to batch ones are less space-consuming and easy to automate, thus usually more desirable in the industry. The shape of our hybrid beads enabled their usage in such dynamic systems without misgiving of column clogging or high pressure drop. Presented in Figure 1, the breakthrough curve of zinc(II) ions sorption on studied biosorbent has no specific, sharp breakpoint and the Zn(II) concentration in effluent increases slowly to achieve its value in inlet (c/c_0_ = 1). The limits for Zn(II) in wastewater, which may be introduced into environment, usually vary between 1.5 and 2.61 mg/L [8,9,10]. The breakthrough point in this work was then assumed as volume of the effluent, when the Zn concentration in effluent reaches 2 mg/L (limit given by legal regulations in Poland [9]). This was marked in the figure as a horizontal line at c/c_0_ = 0.067 (since initial (c_0_) and limit (c_B_) concentration equaled 30 mg/L and 2 mg/L, respectively). This breakthrough point was used in all further experiments as end point of sorption experiment. 

**Figure 2:**
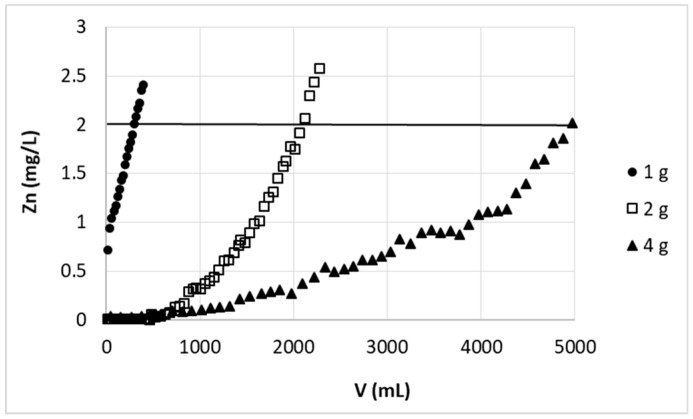
Effect of bed height on zinc(II) ions removal on hybrid pectin-based biosorbent. Mass of sorbent (dry form, fine grain): 1 g (2.9 cm), 2 g (5.7 cm), or 4 g (11.2 cm); flow rate: 60 mL/h; initial concentration of Zn: 30 mg/L; pH = 6.

It is clear that the increase in the bed height increased the number of active sites for the binding of metal ions; the breakthrough point thus is achieved after a longer time or greater volume of effluent. This dependency is however not always proportional and the relationship between bed height change and received ratio of its breakthrough volume or breakthrough time (i.e., (h_2_/h_1_):(V_2_/V_1_) or (h_2_/h_1_):( t_2_/t_1_ ) may be as well < 1 [26,27]; > 1 [28] or equal 1 [29]). In our case the amount of purified solution increased significantly (~6.5-fold) with increasing bed height from 2.9 cm to 5.7 cm (V: 320 mL and 2069 mL, respectively) (Figure 2), giving the ratio of (h_1_/h_2_):(V_1_/V_2_) definitely higher than 1. Increasing in bed height (from 5.7 cm to 11.2 cm) resulted in further increasing of breakthrough point volume, but this growth is not as spectacular as previously—an almost double increase of sorbent in the column resulted in app 2.5 times more purified solution (the ratio (h_2_/h_3_):(V_2_/V_3_) is still higher, but close to 1). This specific behavior may be due to fact that the lowest applied bed height was very close to minimum bed depth (estimated from BDST model to be 2.19 cm)—minimum bed height, which in studied conditions (c_0_ = 30 mg/L, F = 33.97 cm/L), is able to purify solution to established breakthrough concentration (c_B_ = 2 mg/L). Experiments with lower bed height gave unsatisfactory results, e.g., sorbent bed of 2.9 cm height was not sufficient to complete zinc ions removal (see Figure 2)—the most purified fraction still contained 0.72 mg/L of zinc. Increasing the bed height resulted in deeper purification of initial portions of effluent (Zn concentration below 0.01 mg/L). As mentioned previously, as the bed depth increases, the amount of biosorbent binding sites and simultaneously contact time of sorbent with Zn(II) ions increases, which resulted in higher removal efficiency. 

Received results were used for determination of BDST (Bed Depth Service Time) model parameters which were further used for some results prediction.

Applying the equation 1 and using the data from Figure 2 (t—calculated as breakthrough point volume V (mL) divided by flow rate (60 mL/h), Z) the dependence t = a × Z + b was presented as linear curve of equation: t = 9.2636 Z −20.251 (r^2^ = 0.9981). Slope (a) and intercept (b) of the curve with studied conditions (c_0_, F, c_t_ = c_B_) enabled calculation of N_0_ and K_a_ parameters to be 9442 mg/L and 0.0043 L/(mg × h), respectively (Table 1). 

The minimum bed depth necessary for purifying the solution to zinc concentration 2 mg/L in the effluent in studied conditions i.e., Z_0_ calculated for t = 0, was obtained as 2.19 cm (Table 1). 

Following the study of Han et al. [26], changing the flow rate, but keeping all other conditions constant, the slope constant (a) for a different linear flow rate (F) can be directly calculated from the relation:

a’ = a × F/F’(2)

where a and F are the old slope and influent linear flow rate, respectively, and a’ and F’ are the new slope and influent linear flow rate, respectively. 

**Figure 3:**
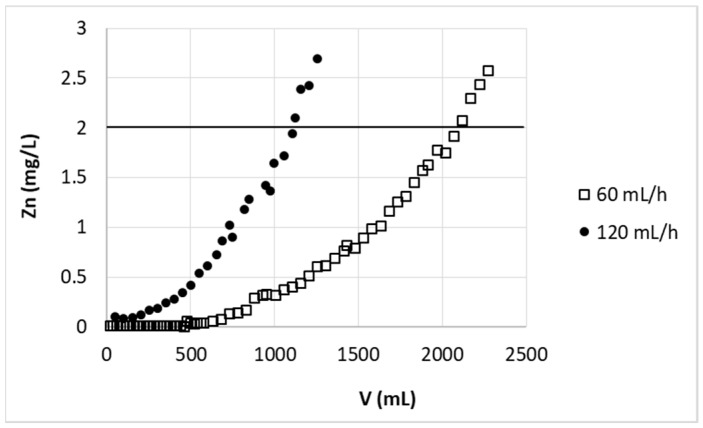
Effect of flow rate on zinc(II) ions removal on hybrid pectin-based biosorbent. Mass of sorbent (dry form, fine grain): 2 g (5.7 cm); flow rate: 60 mL/h or 120 mL/h; initial concentration of Zn: 30 mg/L; pH = 6.

The breakthrough point expected for bed height 5.7 cm and flow rate of F’ = 67.95 cm/h (v = 120 mL/h) equals to t = 6.15 h (or V = 738 mL). Experimental data for that flow rate and bed height 5.7 cm (2g of sorbent) are presented in Figure 3. The real breakthrough point calculated from these data for 120 mL/h equals to t = 9.20 h (calculated as volume 1104 mL divided by flow rate 120 mL/h) (Table 1). From the experimental results shown in Figure 3, it is possible to notice that the two-fold increase of the flow rate (from 60 to 120 mL/h) resulted in double drop of purified solution volume. It is known that at higher flow rate the external film mass transfer diffusion resistance and residence time of adsorbate in column decrease. At higher flow rate the adsorbate leaves the column much before the adsorption equilibrium [26] and whole adsorption ability of the biosorbent cannot be utilized. Additionally, at higher flow rate, thus at lower residence time of adsorbate in column, the intraparticle diffusion becomes less effective [28], which could be the reason of error of above breakthrough point estimation at higher flow rate based on BDST model. The BDST model ignores the intraparticle mass transfer resistance and neglects the external film resistance. It is based on the assumption that the rate of surface reaction between metal ions and the adsorbent is limiting step [22]. The obtained results proved that in this case the intraparticle diffusion influences the zinc(II) ions sorption on pectin-based biosorbent. 

**Figure 4:**
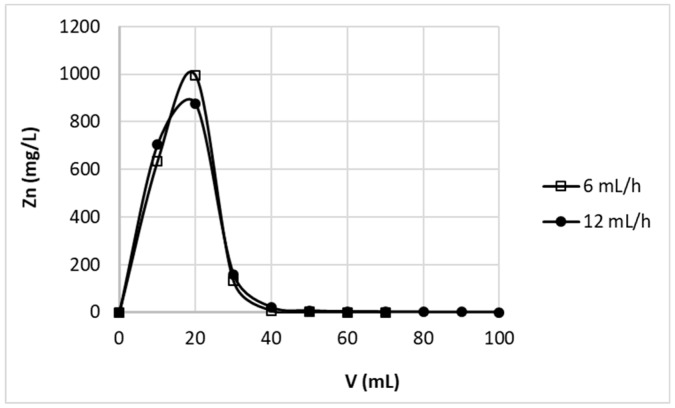
Effect of flow rate on zinc(II) ions stripping from hybrid pectin-based biosorbent. Mass of sorbent (dry form, fine grain): 1 g; flow rate: 6 mL/h or 12 mL/h; stripping solution: 0.1 M HNO_3_.

The striping of metal ions from sorbent is one of the most important issues, which prejudges the possibility of sorbent reuse. The main aim of this process is desorption of previously adsorbed elements to possibly the highest concentrate without sorbent degradation. Desorption should be complete and as little as possible of gentle eluent should be used. Our previous batch studies shown similar efficiency of various 0.1 M (in conversion to H^+^ concentration) acid solutions (0.1 M HCl, 0.1 M HNO_3_, 0.05 M H_2_SO_4_) in removal of zinc(II) ions from studied pectin-based biosorbent [24]. Application of these solutions allowed fast and almost complete (above 95%) removal of zinc ions sorbed on studied material in batch studies. For column desorption studies 0.1M solution of nitric acid was selected. Figure 4 shows the elution curves obtained for bed height 2.9 cm and two various flow rates 6 mL/h and 12 mL/h. The results did not differ with flow rate change, therefore it may be stated that in studied conditions the residence time of protons in the column was sufficient to achieve equilibrium of Zn^2+^–H^+^ exchange. Complete elution of zinc ions was achieved with first 40 mL of the effluent, wherein most of the zinc is removed with first 30 mL of the effluent. 

**Figure 5:**
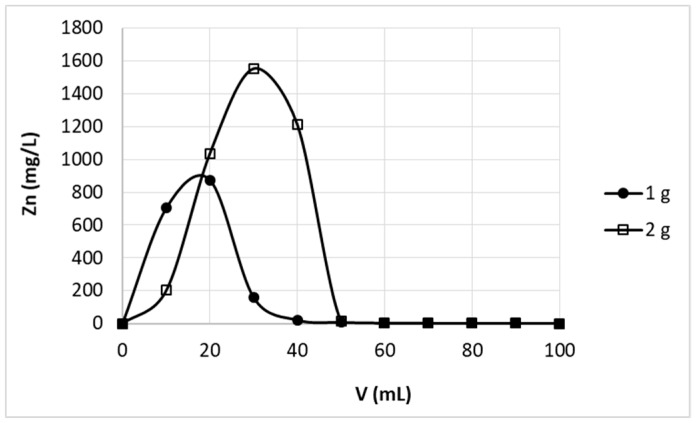
Effect of bed height on zinc(II) ions stripping from hybrid pectin-based biosorbent. Mass of sorbent (dry form, fine grain): 1 g or 2 g; flow rate: 12 mL/h, respectively; stripping solution: 0.1 M HNO_3_.

In the case of higher bed height (5.7 cm) a little more eluent (50 mL) was necessary for complete zinc removal (Figure 5). Taking into account that in studied conditions (c_0_ = 30 mg/L, flow rate 60 mL/h) 2069 mL of solution may be purified to desired level, it may be calculated that zinc ions were concentrated above 40 times in one sorption–desorption step. The time required for performing mentioned concentration of zinc(II) ions mainly consists of sorption step (~34.5 h); desorption step takes ~4 h. Therefore it may be stated that in this case sorption is the step, which prejudges the time needed for concentration of zinc(II) ions. Different dependence may be observed, when the faster flow rate (120 L/h) in sorption step was applied. In this case 1104 mL of purified solution was obtained in 9.2 h. That means 22 fold concentration of zinc(II) ions in about 13.5 h was achieved. In this case duration of both sorption (9.2 h) and desorption (4 h) step was significant. 

**Figure 6:**
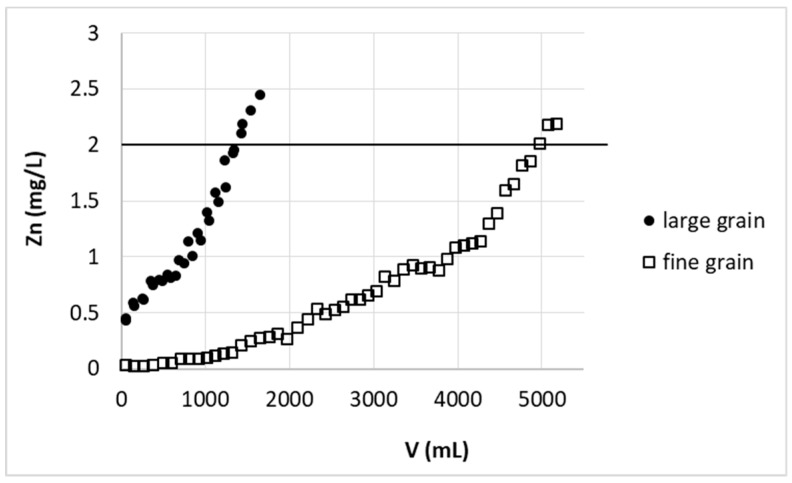
Effect of grain size on zinc(II) ions removal on hybrid pectin-based biosorbent. Mass of sorbent (dry form): small grain 4 g (11.2 cm), large grain 5 g (11.2 cm); flow rate: 60 mL/h; initial concentration of Zn: 30 mg/L; pH = 6.

As mentioned above for the large-scale study, due to some technical problems, sorbent in the form of greater beads was prepared. The removal of zinc(II) ions on this biosorbent was compared with data received for biosorbent of fine grain (Figure 6). 

Unfortunately, because of the more than 2-fold increase in bead dimensions, a 3.7 times decrease in the amount of solution purified was required to reach the desired limit. To explain this behavior in detail further research is needed, which is not the subject of this manuscript, but we roughly expect that it is associated with an increase in the importance of intramolecular diffusion in sorption on large grains. The most accessible external surface of the beads is lower for the same bed volume of larger grain sorbent.

**Figure 7:**
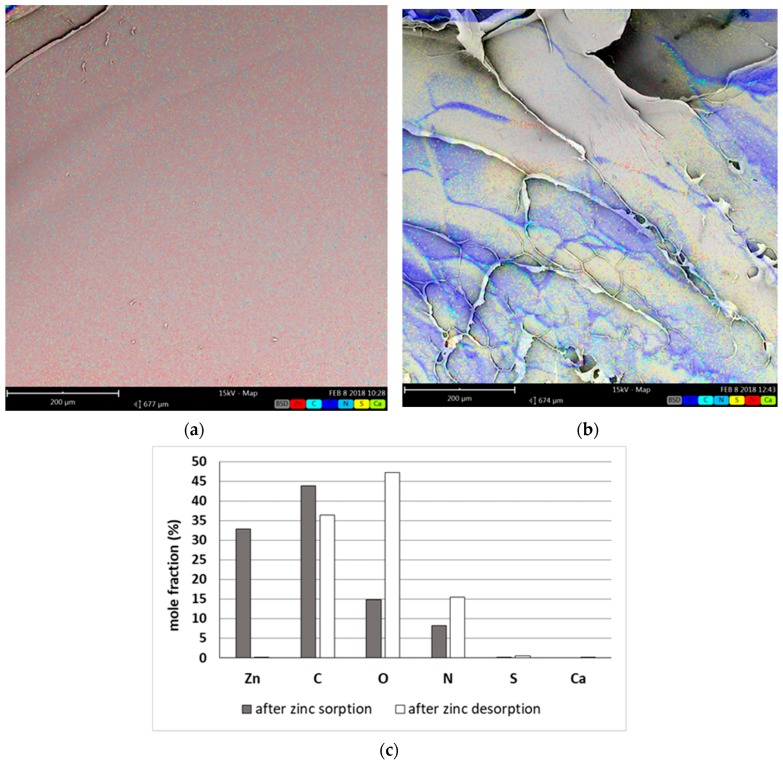
The results of the SEM with energy-dispersive X-ray spectroscopy (EDS) analysis of the biosorbent grain cross-section: (**a**) after zinc sorption (FOV: 677 µm, Mode: 15 kV—Map, Detector: BSD Full); (**b**) after zinc desorption (FOV: 674 µm, Mode: 15 kV—Map, Detector: BSD Full); and (**c**) numerical values of data presented in the pictures (a,b).

To find out if Zn^2+^ are removed due to adsorption on external or internal surface of bead we have applied the EDS analysis (Figure 7). The swollen beads after sorption and desorption processes were cut in half, lyophilized and next corresponding elements (zinc, calcium, and main sorbent elements—carbon, oxygen, nitrogen, and sulfur) distribution was studied using EDS. 

Zinc distribution after sorption was rather uniform throughout the cross-section (Figure 7a), therefore it may be stated that sorption of zinc occurred in grain whole volume i.e., both external and internal surface of bead took part in zinc(II) ion removal. The analysis of grain after desorption (Figure 7b,c) proved on the other hand the completeness of zinc(II) ion stripping—the content of zinc in the grain was close to zero. The latter has been further confirmed by sorption–desorption cycle mass balance (see Table 2).

**Figure 8:**
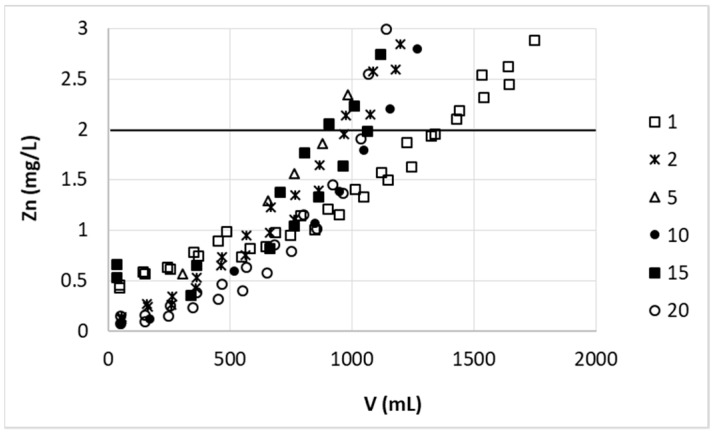
Effect of sorption–desorption cycle number on zinc(II) ions removal on hybrid pectin-based biosorbent. Mass of sorbent (dry form, large grain): 5 g (initially 11.2 cm); flow rate: 60 mL/h; initial concentration of Zn: 30 mg/L; pH = 6.

Successful desorption allowed for study of lifetime of the pectin-based biosorbent. Although it is very important issue, which determines the biosorption process economics and ecological profits, such studies are rarely found in literature, and if done, usually performed only for few cycles [22,30]. In this study, 20 consecutive sorption–desorption cycles were performed on sorbent of large grains prepared in large-scale and using 0.1 M nitric acid solution as stripping agent. Results coming from a few cycles (including the first and the last one) are presented in Figure 8. 

After the first cycle some drop (of approximately 25%) in zinc removal capabilities of our biosorbent occurred, but in the next 19 cycles no further decrease was observed, just some scatter of results. The initial drop in zinc removal efficiency could be due to the fact that during the first sorption the sorbent was in Ca^2+^ doped form (calcium salt was used to prepare the sorbent) and starting from the second cycle it was in H^+^ form what was the aftermath of desorption with acid solution. Therefore no significant pH change could be observed in the first case in opposite to the second and next sorption steps where the Zn(II) ions were exchanged on H^+^ ions. In these cases the pH of the solution during the sorption step decreased from 6 (inlet) to even 3.3 (outlet). Although our previous studies [25] showed no significant differences between sorption capacity of pectin-guar gum biosorbent from solutions of pH 6 and 3, a significant drop in sorption efficiency occurred only when pH dropped from 3 to 2. Since 3.3 was the final pH in the effluent, local pH decrease in some part of column could be greater, influencing the zinc sorption on studied material. Additionally, although no damage or shape changes of H^+^ form sorbent was observed during these 19 cycles, the sorbent grains seemed to be of lower density so they were less pressed in the column. This made more space between the grains and caused bed height increase of ~20%. From one side the residence time of zinc(II) ions in the sorbent bed increased and could enhance the sorption, but on the other side the diffusion of ions into the surface of the sorbent could play more significant role than in the case of tightly packed biosorbent and efficiency of sorption could decrease. The changes in arrangement of pectin-based beads in the columns could be a reason of scatter of results obtaining for the same cycle number and two parallel columns (Figure 8). The most important finding from this experiment was that a lot of (at least 20) sorption–desorption cycles could be performed on one portion of pectin-based sorbent without loss of its effectiveness (if counted from the second cycle). This prompts to further studies on lifetime of the biosorbent as an important factor to judge economic and proecological profitability of the process, on the other hand, it allowed for planning the large-scale tests using the same portion of sorbent. 

Additionally some interesting observations were made—the sorbent after desorption (in H^+^ form) kept the shape and mechanical strength, despite the lack of calcium ions as binding agent, what was confirmed by SEM EDS analysis (Figure 7b,c). The explanation of this phenomenon will be the topic of separate research.

The mass balance of sorption–desorption cycles presented in Figure 8 was also prepared (Table 2). A good recovery (97.8–102.8%) of the element confirmed the stripping method to be applicable for Zn(II) removal from studied biosorbent. In some cases sorption step was performed longer than shown in the Figure 8, therefore the amount of sorbed zinc may vary from cycle to cycle. 

### 3.3. Column Studies—Large-Scale

The aim of the large-scale studies was to confirm the efficiency of zinc(II) ions sorption on studied biosorbent in semipilot scale and additionally check if phenomena, which could not be observed in laboratory scale and may influence the sorption effectiveness. Including, for instance, the aspect of bead compression that could prevent the desired flow rate achievement, especially during drop-feed column work [23].

Our biosorbent was in the form of round beads (diameter ~3.5 mm in wet form), so if only mechanical strength was adequate the compression should not happen. Other threat concerning also mechanical strength of our beads was their stability in the conditions of higher flow rates, comparable with those applied in the industry. Generally during all large-scale studies no problems with high flow rate (even 200 L/h (442.5 cm/h)) were observed, independently on drop-fed or reverse-fed column work. Also no significant pressure drop on our bed occurred. The beads did not change their shape or volume during the experiments, therefore it was confirmed that they are suitable for industrial application from the mechanical strength point of view. 

**Figure 9:**
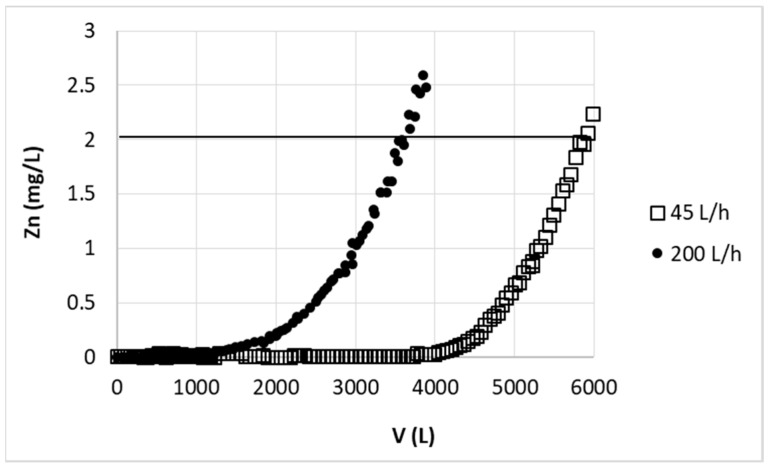
Effect of flow rate on zinc(II) ions removal on hybrid pectin-based biosorbent. Mass of sorbent (dry form, large grain): 16 kg; initial concentration of Zn: 30 mg/L; pH = 6.

The efficiency of zinc(II) ion sorption in large-scale was first tested using two various flow rates and model zinc solution. The first applied flow rate (200 L/h, 442.5 cm/h) was roughly and simply calculated by multiplying the linear flow rate from small scale (33.97 cm/h) by the same enlargement as bed height increase (11.2 cm to 120 cm). The linear flow rate should equal to 363.5 cm/h and taking into account new dimension of column (240 cm) flow rate should be 164 L/h. However since in wider column beads of sorbent are better packed than in the case of narrow laboratory scale column (16 kg of biosorbent packed into column occupied about 54 L instead of 62 L, as calculated form the proportion of 5 g:19.43 mL to 16 kg:62 L), thus amount of active sites per centimeter of bed height in large column is greater than in the case of laboratory scale column. Therefore the calculated flow rate was increased to 200 L/h. As the result 3600 L of the solution purified to desired breakthrough point (2 mg/L) was obtained (see Figure 9). Taking into account that in this case sorption parameters (bed height and linear flow rate) were proportionally enlarged from small scale, simple proportion was used to compare result from laboratory and large-scale: using 5 g of sorbent average volume of purified solution (cycle 2–20) was ~0.95 L, using 16 kg it should be 16000 × 0.95/5 = 3040 L. The experimental results were then almost 20% better than estimated. Additionally it should be noted that points obtained in two consecutive experiments overlapped with each other, thus the scatter of the results was significantly lower than during the laboratory studies. The enhancement in zinc sorption efficiency and in results repeatability could be due to more stable arrangement of beads in the column as well as smaller space between the grains, so the external diffusion of ions from the solution into the sorbent surface took less time. 

Reduction of the flow rate to 45 L/min caused, that the amount of solution purified to the desired concentration of zinc (2 mg/L) increased to 5900 L (Figure 9). Reduction of the flow rate, and thus extending the process time, increased the problem with bubbles of air in the column. This important observation was enabled thanks to the column transparency. 

**Figure 10:**
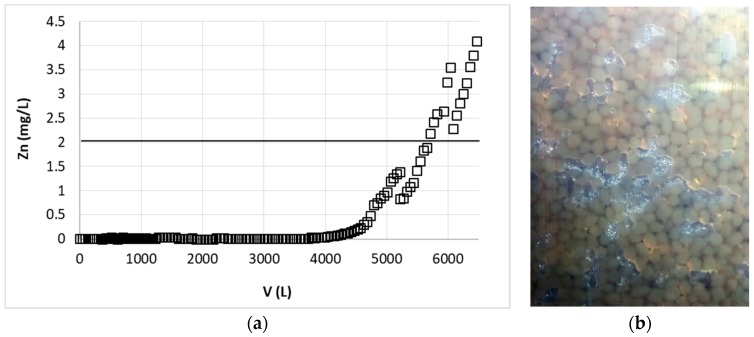
(**a**) Effect of zinc(II) ions removal on hybrid pectin-based biosorbent in the presence of air bubbles in the column. Flow rate: 45 L/h, mass of sorbent (dry form, large grain): 16 kg; initial concentration of Zn: 30 mg/L; pH = 6. (**b**) the air bubbles in the column.

Generally the bubbles of gas appeared during all experiments (Figure 10b); the column was then periodically degassed. Gas bubbles that happened to be bubbles of air (confirmed by gas chromatography), were released through vent valves on the top of the column by tapping the column from down to up. Formed air bubbles partially blocked the access to the surface of the biosorbent causing slight decrease of the sorption efficiency, after degassing the efficiency could return to normal level. This may be observed in Figure 10a, e.g., from app 5000 L of effluent the process was performed in the presence of great amounts of air bubbles till about 5200 L when the concentration of zinc in the effluent equaled 1.4 mg/L. After removal of gas from the column the concentration of zinc in effluent decreased to 0.8 mg/L and the process was continued to regassing of the column. At ~6000 L and zinc concentration 3.5 g/L the column was again degassed and the concentration of zinc decreased to 2.3 mg/L, thus the sorption efficiency increased. Therefore this issue is important to be taken into account when industrial application is considered. The solution may be the periodical vibration of the column with automatic vent valves system. There are also some industrial solutions for removal of microbubbles from treated solution before it reaches the device (e.g., heat exchanger or column). Our sorption station was equipped with microbubbles gas separator, it was the next device after the peristaltic pump, however it did not prevent gas release from solution in the column. This could be due to the fact that air was not in the form of microbubbles, but it was dissolved in the solution. Gas dissolution process could be promoted by pumping the cold water into our IBC containers. Even though during slow warming to room temperature the solution of air in Zn(II) solution could become supersaturated, we had no technical possibilities to remove the air from solution in the 1000 L container. The solubility of air in the aqueous solution, beside the temperature and pressure, also depends on the composition of solution. Additionally the factors such as vibes or stirring (or other kinetic distortion of solution) may also induce the gas release especially from saturated or supersaturated solution. Conditions such as slight temperature increase, solution composition and pH changes, pressure drop, and kinetic distortion of the solution (pumping, flow through the elbows, nozzles, and between the sorbent grains) occurred in the column during the sorption and desorption processes and could induce generation of the air bubbles. 

To check the possibility of the large-scale result prediction by the BDST model appropriate calculations were made. Due to the change of sorbent grain size some coefficient had to be introduced, therefore the value of breakthrough volume calculated from BDST model was divided by 3.7 as it was found during the comparison of sorption on fine and large grain. Using mentioned coefficient, bed height Z = 120 cm and keeping the other parameters unchanged (c_0_ = 30 mg/L, c_t_ = c_B_ = 2 mg/L), calculated breakthrough volumes for new linear flow rate F’’ and F’’’ (99.6 cm/h and 442.5 cm/h, respectively), was found to be 4361 L and 3519 L for flow rate of 45 L/h and 200 L/h, respectively. These results did not take into account that experiments were conducted on sorbent in H^+^ form instead of Ca^2+^, the proper results should be then decreased of ~25% to 3276 L and 2639 L, respectively. After such computations the results are significantly lower than experimental ones (5900 L and 3600 L), but as mentioned earlier BDST model neglect some processes responsible for sorption efficiency. Additionally introduced coefficients of grain size and sorbent form could be a source of some errors. 

In desorption studies two acids were used—0.1 M nitric acid, as the most commonly used for stripping of various elements, and sulfuric acid of the same proton concentration (i.e., 0.05 M H_2_SO_4_). The selection of acid solution should depend on further management of solution from desorption process. The sulfuric acid was selected as alternative to nitric one due to possibility of further recovery of zinc from sulfate solution by electrolysis. The electrolysis of zinc from nitric acid solution is not effective. Additionally the accepted limit of the sulfate ions in wastewater is higher than for nitrate ones [9], thus sulfuric acid as stripping agent may be desirable.

**Figure 11:**
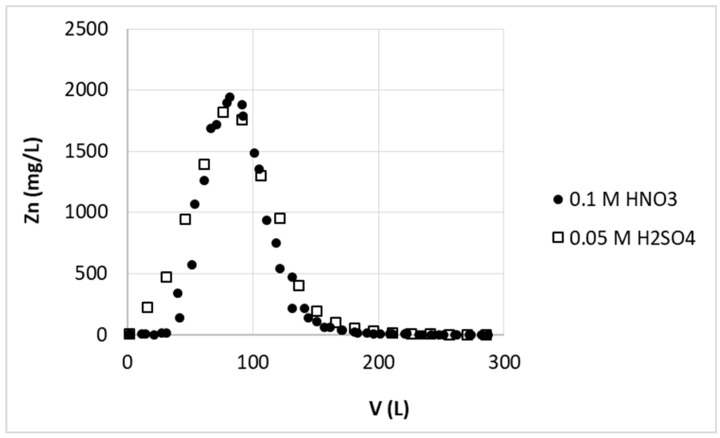
Effect of type of acid on zinc(II) ions stripping from hybrid pectin-based biosorbent. Mass of sorbent (dry form, large grain): 16 kg; flow rate: 30 L/h; stripping solution: 0.1 M HNO_3_ or 0.05 M H_2_SO_4_.

The results shown in Figure 11 for these acids do not differ significantly—in both cases fast and complete removal of zinc from biosorbent are illustrated by sharp and symmetric peak of elution curve. Taking into account that 5900 L of the solution was purified to desired level and only 120 L of acid solution was used for zinc(II) removal from biosorbent, the zinc(II) ions were concentrated 49 times in one sorption–desorption step. This should allow for further processing of the solution and metal recovery by e.g., electrolysis process.

Satisfactory results of large-scale studies on sorption from model solution inclined us to test the zinc removal from real wastewater from galvanizing plant. The wastewater from galvanizing plant has various amounts of zinc as well as other components diluted. The composition depends on wastewater origin—it could be used plating bath or rinse water from various stage of rinsing. We prepared simulated rinse water by dilution of original weakly acidic bath for zinc electroplating to final Zn concentration 30 mg/L (as in the model solution). This enabled to study the effect of real wastewater components on zinc(II) ions removal on studied biosorbent. In our case the substances were as follows; potassium chloride, boric acid, and a few additional components e.g., brightener, surfactants, etc., which could influence the sorption efficiency.

**Figure 12:**
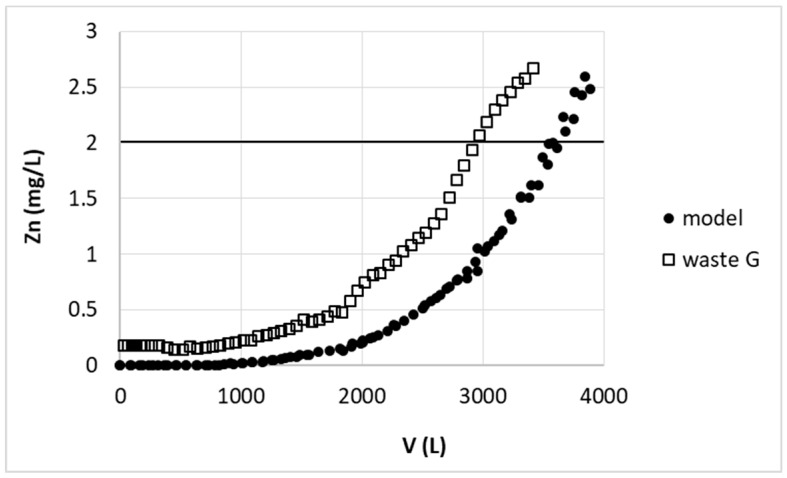
Comparison of zinc(II) ions removal from model solution (model) and galvanizing plant wastewater (waste G) on hybrid pectin-based biosorbent. Mass of sorbent (dry form, large grain): 16 kg; flow rate: 200 L/h; initial concentration of Zn: 30 mg/L; pH = 6.

The comparison of the results of zinc ions removal from model (model) and real (waste G) solutions are presented in Figure 12. Despite of various additional compounds presence, the breakthrough volume for real wastewater was only 17% lower than for model solution. This slight drop in zinc removal efficiency could be due to the fact that substances present in wastewater may act as complexing agent towards zinc(II) ions. These could be among others dextrin, organic acids, aldehydes or polymers with terminal amino groups used as brightener or surfactants like sodium lauryl ether sulfate or dodecanal. Such compounds may partially mask zinc(II) ions and prevent their sorption on pectin biosorbent. Additionally mentioned substances are molecules of rather big size and may clog the access to some active sites of sorbent, thus decrease its sorption capacity. 

However the result of rinse water purification seems to be satisfactory and confirmed possibility of our biosorbent usage in large-scale for real wastewater. Additionally our sorption–desorption station was of size that could be already used in small galvanizing plant.

As a next step, very long studies should be performed to estimate real lifetime of the biosorbent. However our results—20 cycles of sorption–desorption process without loss of sorbent efficiency—are, in the case of biosorbent, of great significance.

## 4. Conclusions

Our small scale studies confirmed that pectin-based biosorbent in the form of beads is suitable for zinc ions removal in column process to the level permitted by law. The effectiveness of the sorption is affected by flow rate, bed height, and grain size. The desorption of zinc using 0.1 M nitric acid solution is fast and complete (~100% recovery) and 40-fold of Zn(II) concentration may be achieved in one sorption–desorption cycle. The most important and successful result is that at least 20 sorption–desorption cycles may be performed on one portion of our biosorbent without loss of its effectiveness (counting from the second cycle). Scaling up 3200 times confirmed that form and mechanical stability of our pectin-based sorbent is suitable for column usage with flow rates used in industry. The results of zinc(II) ions removal in such conditions were even better than estimation based on small scale results. Applying the studied conditions (16 kg of sorbent, flow rate 45 L/h) even 5900 L of solution containing 30 mg/L of zinc could be purified to the limit given by legal regulations (2 mg/L). The desorption with 0.1 M HNO_3_ or 0.05 M H_2_SO_4_ was, as previously, fast and complete and almost 50-fold zinc concentration was achieved. Large-scale studies enabled also important threat observation, which should be taken into account during industrial apparatus designing: if the treated solution is strongly aerated, the bubbles of air may be generated in the column and influence the sorption efficiency. The successful results of zinc(II) ion removal from real wastewater from a galvanizing plant additionally proved our biosorbent to be promising for industrial applications. 

## Figures and Tables

**Table 1:** The values used for determining the parameters of Bed Depth Service Time (BDST) model and values calculated on their basis, t—time of obtaining in the effluent concentration c_t_, Z—bed height, c_0_—initial concentration of zinc(II) ions, F, F’—linear flow rate, c_B_—breakthrough concentration, a, a’—slope of the curve, b—intercept of the curve, N_0_—adsorption capacity, K_a_—rate constant in BDST model, Z_0_—minimum bed depth.

Data Used for Determining the Parameters of Equation t = a × Z + b			
t (h)	5.33	34.48	82.85
Z (cm)	2.9	5.7	11.2
studied conditions			
c_0_ (mg/L)	30		
F (cm/L)	33.97		
c_t_ = c_b_ (mg/L)	2		
F’ (cm/L)	67.95		
calculated parameters			
a = N_0_/(c_0_ × F)	9.2636		
b = −(1/(K_a_c_0_)) × ln(c_0_/c_t_ − 1)	−20.251		
N_0_ (mg/L)	9442		
K_a_ (L/(mg × h))	0.0043		
Z_0_ (cm)	2.19		
a’ = a × F/F’	4.63		
**t (h) calculated for Z = 5.7 cm and F’**	**6.15**		
**t (h) experimental for Z = 5.7 cm and F’**	**9.20**		

**Table 2:** The mass balance of repeated zinc(II) ions sorption–desorption cycles in column studies.

Cycle No.	(1) Zn Introduced to the Column (mg)	(2) Zn in Effluent (Sorption Process) (mg)	(3) Zn in Effluent (Desorption Process) (mg)	(4) the Sum of (2) and (3) (mg)	Recovery (%)
**1**	54.03	2.60	51.83	54.43	100.7
**1’**	50.88	2.14	48.02	50.16	98.6
**5**	41.66	2.92	38.24	41.16	98.8
**5’**	40.45	2.49	37.17	39.66	98.0
**10**	41.82	1.85	39.47	41.32	98.8
**10’**	39.47	1.47	37.42	38.89	98.5
**11**	52.42	3.41	49.76	53.17	101.4
**11’**	50.59	2.88	49.13	52.01	102.8
**14**	37.55	1.38	37.03	38.41	102.3
**14’**	36.47	0.82	34.86	35.68	97.8
**15**	38.09	1.79	36.00	37.79	99.2
**15’**	36.21	1.14	34.58	35.72	98.6
**20**	35.73	1.14	34.97	36.11	101.1
**20’**	33.57	0.78	32.41	33.19	98.9

## Supplementary Materials

The following are available online at http://www.mdpi.com/2073-4360/11/1/96/s1, Video S1: The working apparatus of semipilot scale.
